# Attention Configures Synchronization Within Local Neuronal Networks for Processing of the Behaviorally Relevant Stimulus

**DOI:** 10.3389/fncir.2018.00071

**Published:** 2018-08-29

**Authors:** Eric Drebitz, Marcus Haag, Iris Grothe, Sunita Mandon, Andreas K. Kreiter

**Affiliations:** Center for Cognitive Science, Brain Research Institute, University of Bremen, Bremen, Germany

**Keywords:** visual cortex, macaque monkey, gamma-band, area V4, neuronal network configuration, spatial selective attention, functional coupling, dynamic assembly formation

## Abstract

The need for fast and dynamic processing of relevant information imposes high demands onto the flexibility and efficiency of the nervous system. A good example for such flexibility is the attention-dependent selection of relevant sensory information. Studies investigating attentional modulations of neuronal responses to simultaneously arriving input showed that neurons respond, as if only the attended stimulus would be present within their receptive fields (RF). However, attention also improves neuronal representation and behavioral performance, when only one stimulus is present. Thus, attention serves for selecting relevant input and changes the neuronal processing of signals representing selected stimuli, ultimately leading to a more efficient behavioral performance. Here, we tested the hypothesis that attention configures the strength of functional coupling between a local neuronal network's neurons specifically for effective processing of signals representing attended stimuli. This coupling is measured as the strength of γ-synchronization between these neurons. The hypothesis predicts that the pattern of synchronization in local networks should depend on which stimulus is attended. Furthermore, we expect this pattern to be similar for the attended stimulus presented alone or together with irrelevant stimuli in the RF. To test these predictions, we recorded spiking-activity and local field potentials (LFP) with closely spaced electrodes in area V4 of monkeys performing a demanding attention task. Our results show that the γ-band phase coherence (γ-PhC) between spiking-activity and the LFP, as well as the spiking-activity of two groups of neurons, strongly depended on which of the two stimuli in the RF was attended. The γ-PhC was almost identical for the attended stimulus presented either alone or together with a distractor. The functional relevance of dynamic γ-band synchronization is further supported by the observation of strongly degraded γ-PhC before behavioral errors, while firing rates were barely affected. These qualitatively different results point toward a failure of attention-dependent top-down mechanisms to correctly synchronize the local neuronal network in V4, even though this network receives the correctly selected input. These findings support the idea of a flexible, demand-dependent dynamic configuration of local neuronal networks, for performing different functions, even on the same sensory input.

## Introduction

Successful and goal directed behavior within our complex world requires a dynamic and adaptive processing of relevant information. Thus, local neuronal networks are required to perform different functions depending on the current requirements of information processing. A well-known example for such adaptation of network functions is the attention-dependent selection of different subsets of afferent input for effective processing of relevant visual information. Several studies investigating responses of neurons to simultaneous and convergent input showed that neurons responded almost as if only the attended stimulus would be present within their receptive field (RF) (Moran and Desimone, 1985; Treue and Maunsell, 1996; Luck et al., 1997; Reynolds et al., 1999; Lee and Maunsell, 2010; Grothe et al., 2012). Such changes of network function quickly follow changing behavioral demands although anatomical connections cannot change on such short timescales (Gilbert and Sigman, 2007; Carandini and Heeger, 2012; Park and Friston, 2013). However, recent theoretical work indicates that modulating γ-band synchronization can change the strength of the functional coupling between neuronal populations. Selective synchronization of a local network with only those upstream inputs representing the attended stimulus could therefore constitute a mechanism underlying this attention-dependent bias of neuronal responses (Börgers and Kopell, 2008; Battaglia et al., 2012; Wildie, 2012; Hahn et al., 2014; Harnack et al., 2015; Palmigiano et al., 2017). We and others showed indeed that the strength of γ-band phase coherence (γ-PhC) between V1 and V4 populations with overlapping receptive fields (RF) was much stronger when the V1 population represented the attended instead of the non-attended stimulus (Bosman et al., 2012; Grothe et al., 2012, 2018). These theoretical and experimental results indicate that attention-dependent selective processing of relevant afferent input depends on the strength of functional coupling between neurons along the visual pathway.

Yet, attention also modulates perception and neuronal responses when there is no competition between inputs. With only one stimulus inside the RF, attention has been shown to increase firing rates (Treue and Maunsell, 1996; McAdams and Maunsell, 1999; Reynolds et al., 2000; Wegener et al., 2004), to reduce the response variability of individual neurons (Mitchell et al., 2007; Galashan et al., 2013; Schledde et al., 2017) and to reduce the shared response variability of neuronal populations (Cohen and Maunsell, 2009; Mitchell et al., 2009; Schledde et al., 2017). Thus, besides the selective routing of relevant information, attention also modifies network functions for improving the processing of a single attended stimulus. Such modifications could be obtained by reorganizing the network's internal pattern of functional connections by changing the pattern of γ-band synchronization (Aertsen et al., 1989; König and Schillen, 1991; Singer, 1993; Segev and Rall, 1998; Usrey et al., 2000; Azouz and Gray, 2003; Womelsdorf et al., 2007; Tiesinga and Sejnowski, 2010; Battaglia et al., 2012; Engel et al., 2013).

Based on these considerations, we hypothesized that effective processing of an attended stimulus requires a specific configuration of local neuronal networks in visual cortex. This attention-dependent and stimulus-specific configuration of a local network is established by a specific pattern of γ-band synchronization between neurons processing the attended stimulus. From this hypothesis, we derive three simple predictions: (1) In the presence of multiple stimuli in the population receptive field (pRF) of a local network, the pattern of γ-band synchronization and hence, the corresponding configuration of functional connections should depend on the attended stimulus. (2) If this pattern would indeed be stimulus specific, it should be very similar to the pattern observed, if only the attended stimulus is present. (3) Deviating patterns of γ-band synchronization should reflect erroneous network configurations and go along with deteriorated behavioral performance.

To test the hypothesis, we recorded neuronal activity in area V4, using two closely spaced microelectrodes, while monkeys attended one of two stimuli within the RF. The stimuli were placed within the V4 pRF such that they induced local synchronization and responses of different strength. We found that switching attention between these two stimuli resulted in local γ-band synchronization strength almost as if the attended stimulus would be present without nearby distractor. Behavioral errors were preceded by local synchronization deviating strongly from that observed for successfully executed trials, while spiking activity showed only small differences between successful and wrong task execution.

## Materials and methods

### Surgical preparation

Two male macaque monkeys (*Macaca mulatta*) were implanted under aseptic conditions with a titanium head holder and a recording chamber above area V4. The target area was identified by evaluation of MRI-scans performed before surgery. All procedures were approved by the local authorities (Der Senator für Gesundheit, Bremen, Germany) and were in accordance with the regulation for the welfare of experimental animals issued by the Federal Government of Germany and with the guidelines of the European Union (2010/63/EU) for care and use of laboratory animals.

### Behavioral task

**Figure 1:**
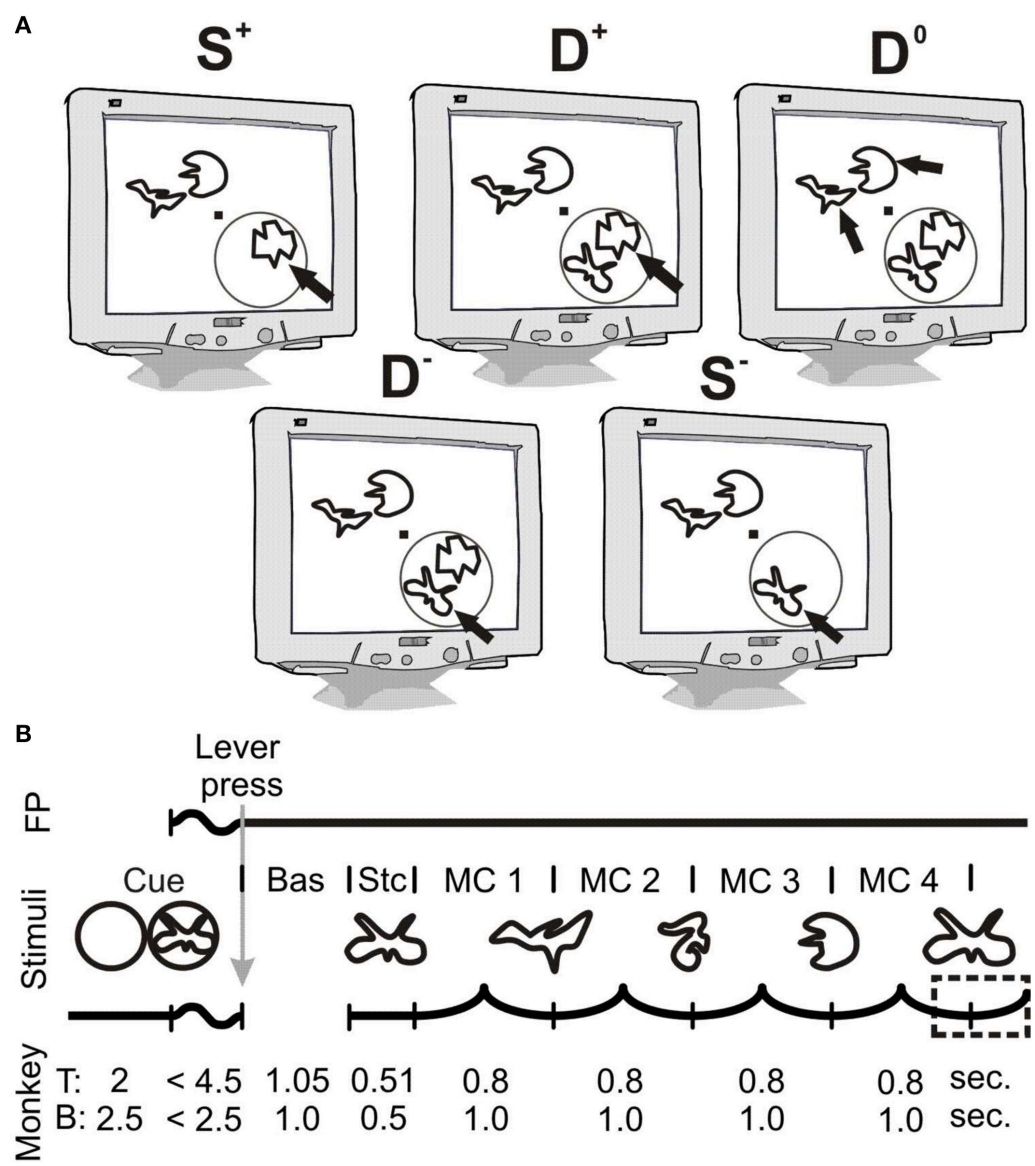
Stimulus configuration and temporal structure of the shape-tracking task. **(A)** Schematic illustration of task conditions. Black arrows indicate the cued target stimulus; other stimuli serve as distractors. The RF of the recording site in V4 is depicted as a gray circle, which does not appear on the display. Trials could contain either one or two stimuli in the V4 RF. The abbreviations indicate the number of stimuli within the RF (“S” for single and “D” for double presentation in the V4 RF) and the direction of attention to either the stimulus inducing higher “^+^” or lower values “^−^” of spiking activity or γ-synchronization. The condition with two stimuli in the RF but attention directed to stimuli in the opposite hemi-field is referred to as D^0^ (Double Attend Out). **(B)** Temporal structure of the shape-tracking task. During the cueing period, a spatial cue was present on screen. The cue was a simple ring for monkey T and a ring enclosing the upcoming target shape for monkey B. After appearance of the fixation point (FP), monkeys started fixation and initiated the trial by pressing a lever. Simultaneously, the spatial cue disappeared (faded within 200 ms for monkey B). After a baseline period (Bas.), followed by static presentation (Stc.) of the initial shapes at each location, they morphed through a sequence of different shapes until the initial shape at the cued location reappeared. Monkeys had to signal this reappearance within a response period indicated by the dashed rectangle. Bas, baseline period; Cue, Cueing period; FP, fixation point; MC, morph cycle; Stc, static presentation period.

The animals performed a highly attention-demanding shape-tracking task. In the following, for task parameters differing between individuals, the parameters for monkey T are mentioned in the text and those for monkey B follow in brackets. Visual stimuli were presented on a 20-inch CRT-monitor with a resolution of 1024 × 768 pixels (1152 × 864 pixels) and a refresh rate of 100 Hz. The screen was placed 90.5 cm (92 cm) in front of the monkey that was sitting in a custom-made primate chair. Visual stimulation comprised a fixation point and up to four simultaneously presented complex shapes (Figure 1A). Figure 1B shows the sequence of stimuli and events of a single trial: It starts with the appearance of a spatial cue, which indicates the position of the behaviorally relevant stimulus in the upcoming trial. During this period of the trial animals were allowed to move their eyes freely. The spatial cue consisted of a 1° (1.5°) diameter ring with a linewidth of 0.04° (0.075°) centered over the position of the upcoming target stimulus. For monkey B, the cue contained in addition the initial shape of the upcoming trial, because it helped to increase performance during training of the task. After 2.0 s (2.5 s) a central 0.15° × 0.15° fixation point (FP) appeared, which required the animals to start fixation and subsequently to initiate the trial by pressing a lever inside the primate chair within 4.5 s (2.5 s). Following trial start, the spatial cue disappeared (faded within 200 ms) and a baseline period of 1050 ms (1000 ms) began. Subsequently the static presentation period started with the appearance of three or four differently shaped stimuli, all at the same eccentricity between 2.5° and 3.5° (2.1°-2.5°) of visual angle (Figure 1A). Either one or two adjacent stimuli were located in the lower visual field quadrant contralateral to the recording sites in area V4. The other two stimuli appeared at positions mirrored across the FP in the upper, ipsilateral visual field quadrant. The stimuli presented at each of the four positions differed in color (red, green, yellow, blue; luminance: 3.7–5 cd/m^2^, background luminance: 0.03 cd/m^2^). The assignment of these colors to the four stimulus positions was constant throughout a recording session. Stimuli at all locations could serve as target. The initial complex shapes at each stimulus location were presented statically for 510 ms (500 ms) and subsequently started to morph continuously into other complex shapes (see also: Taylor et al., 2005; Grothe et al., 2012). A single morphing cycle (MC), i.e., morphing completely from one shape into another shape, lasted 800 ms (1,000 ms). Trials consisted of two to four MCs. All shapes were taken randomly with equal probability out of a set of 8 shapes (6 shapes). The reappearance of the initial shape at the cued stimulus location required the monkeys to release the lever within a time window ranging from 310 ms before the shapes' complete reappearance to 400 ms afterwards (−350 ms to 150 ms; Figure 1B, dashed rectangle). The appearance of the targets' initial shape within the sequence of MCs at the distractor locations or the distractors own initial shape had to be ignored. For monkey T, all 8 shapes could become initial shape, whereas for monkey B, the initial shape of the target stimulus was always the same, within and across sessions. Throughout the whole trial, the eye position was monitored by video-oculography (monkey T: I Scan Inc., Woburn, MA, USA; monkey B: custom-made eye tracking system) and the direction of gaze was not allowed to deviate from the FP by more than 0.5°. If monkeys released the lever within the response window, they were rewarded with a small amount of diluted fruit juice. If they broke fixation or responded outside the response window, trials were aborted without reward.

### Recording procedure

Simultaneous intracortical recordings in the upper layers of visual area V4 were performed using two epoxy-insulated tungsten microelectrodes (1–3 MΩ, shank diameter 125 μm FHC Inc., Bowdoin, ME, USA; 330 μm distance between shanks). The electrode signals were amplified 4000x (1000x) (monkey T: 4x by a wideband preamplifier MPA32I and 1000x by a PGA 64, 1-5000 Hz, both Multi Channel Systems GmbH, Germany; monkey B: same setup but gain factor 10 for preamplifier and 100 for PGA) and digitized with 25 kHz sampling rate and 12 bit (16 bit) ADC resolution. The reference electrode for monkey T was the recording chamber, a titanium cylinder of 25 mm diameter implanted into the bone and touching the dura. The electrode signals of monkey B were referenced to a low impedance electrode (< 0.1 MΩ), positioned on top of an epidural array (contacting the bone), placed above area V1. Before recordings, the pRF for each recording site was mapped manually as the minimal response field based on multi-unit- and LFP-responses, while the animals performed a fixation task. Both microelectrodes were placed such that the recorded neurons shared major parts of their pRFs. Locations and colors for the two stimuli within the overlapping pRFs were chosen such that they caused responses of different strength.

### Data analysis

Customized scripts for Matlab (version R2013a, MathWorks, Natick, MA, USA) were used for all offline data analysis procedures described below. Data were analyzed for the spiking activity of a small group of neurons by calculating the entire spiking activity (ESA). As a measure for the strength of functional coupling, we calculated the PhC between two signals, either representing the overall local population activity (LFP) and the activity of a small group of neurons (ESA-LFP PhC) or the activity of two separate groups of neurons of the same local network (ESA-ESA-PhC). For analysis of spiking activity, we used the ESA-signal because it is more sensitive in detecting neuronal responses in data with low signal to noise ratio, since it does not reject sub-threshold events. Furthermore, the independence from thresholding provides the advantage of integrating over all spikes (even small ones) of a population, resulting in a more complete estimate of the actual population response. The ESA of neurons near the recording electrode's tip (50 μm radius according to Brosch et al., 1997) was obtained by band-passing the raw signal using a FIR-filter between 0.3 and 12.2 kHz in forward and backward direction (to avoid phase shifts). Subsequently, the band-limited signal containing the spiking activity was full-wave rectified and low-pass filtered (forward and backward) at 160 Hz and down-sampled to 1 kHz (Legatt et al., 1980; Frien et al., 2000). As opposed to standard multi-unit activity, this procedure delivers a continuous instead of a binary signal, which is known to represent the spiking activity of multiple neurons surrounding the electrode tip (Supér and Roelfsema, 2005). ESA-responses were obtained by subtracting for each recording site the mean spontaneous activity from the ESA-values recorded during the analysis period. Spontaneous activity was estimated as the average ESA taken from 150 ms (250 ms) after the baseline period started to its end over all correctly performed trials of a recording session. Note, that during baseline period (Figure 1B) no visual stimulus but the FP was present on screen. The LFP was obtained from the recorded signal by low-pass filtering with a FIR-filter (−3 dB point at 170 Hz) in forward and backward direction and subsequent down-sampling to 1 kHz.

The time-frequency decomposition of ESA and LFP signals was performed by convolving the signals with complex Morlet's wavelets $\omega (t,f_{0})=A\exp(-t^{2}/2\sigma_{t}^{2})\;\exp(2i\pi f_{0}t)$, with $\sigma_{f}=\frac{1}{2\pi \sigma_{t}}$. Morlet's wavelets have a Gaussian shape both in time (SD: σ_*t*_) and frequency dimension (SD: σ_*f*_) and were normalized such that their total energy was 1. The normalization factor *A* was defined as:

(1) $$A=(\sigma_{t}{\sqrt{\pi )}}^{-0.5}.$$

Central frequencies *f*_0_ of the Morlet's wavelets ranged from 5 to 160 Hz according to the scheme described by Torrence and Compo (1998) with a ratio of $\frac{f_{0}}{\sigma_{f}}=6\;$(Tallon-Baudry et al., 1997; Taylor et al., 2005). The wavelet transform provides complex coefficients ${\tilde{\;x}}_{j}^{r}\;$for electrode *j* and trial *r* at time *t* and frequency *f*, which can be expressed as their amplitude *A* and phase Φ:

(2) $$\begin{array}{r} {\tilde{x}}_{j}^{r}\left(t,f\right)=\;A_{j}^{r}\left(t,f\right)e^{i\Phi_{j}^{r}\left(t,f\right)} \end{array}$$

The frequency-dependent power of LFP and ESA-signals was computed by taking the square of the absolute value of the convolution's result and dividing it by the Nyquist frequency (500 Hz). The phase component for each time and frequency bin was used for estimation of PhC over *N* trials, between electrodes *j* and *k*, as follows (see also: Lachaux et al., 1999; Grothe et al., 2012):

(3) $$\begin{array}{r} PhC\left(t,f\right)=|\frac{1}{N}\sum_{r}e^{i\Phi_{j\;}^{r}\left(t,f\right)-i\Phi_{k}^{r}\left(t,f\right)}| \end{array}$$

The PhC was calculated for each electrode pair, thus delivering one ESA-ESA measure and two ESA-LFP pairs (as both sites of the pair can deliver either the ESA or the LFP) for PhC analyses. ESA and LFP signals were never taken from the same electrode to avoid the possibility that the same spike contributed to ESA and LFP. PhC values were bias-corrected by subtracting the expected value (EV) for the PhC, as estimated from the same number trials (*N*) with random phase relations (Grothe et al., 2012).

(4) $$\begin{array}{r} EV\left(N\right)=\;\frac{\sqrt{\pi }}{2\sqrt{N}} \end{array}$$

**Figure 2:**
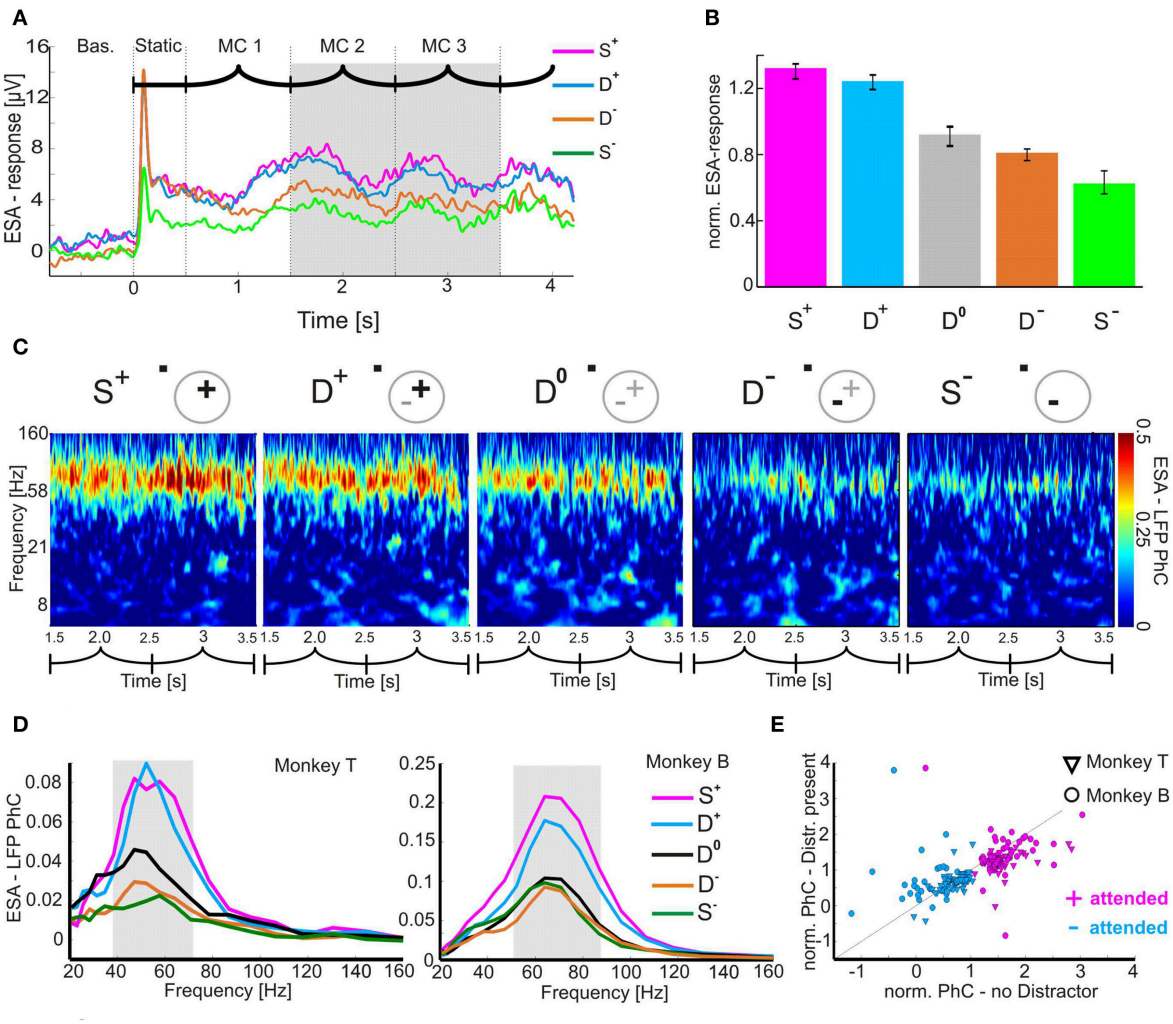
Attention-dependent modulation of neuronal responses and their coupling to the local population activity. **(A)** Time course of mean ESA-responses during the shape-tracking task of an example case (visual stimulation sequence indicated above as in Figure 1). For clarity, the D^0^-condition is omitted. **(B)** Histogram of normalized median ESA-responses during MC 2 and 3 (highlighted gray in **A**) and pooled over both animals. Error-bars indicate 95 % confidence intervals. **(C)** Time-frequency plots of the PhC between ESA and LFP for all task conditions during MC 2 and 3 of an example case. Pictograms indicate stimulus configuration within the V4 pRF. Plus and minus signs depict the stimuli inducing stronger and weaker γ-PhC, respectively. Bold highlighted signs correspond to the attended stimulus. (**D)** Median spectra for ESA-LFP PhC of both animals. The individual γ-bands are highlighted in gray. **(E)** Scatter-plot of normalized ESA-LFP PhC (median over MC 2/3 and the individual γ-band) for conditions without vs. with distractor in pRF.

For the analysis of PhC and power in the γ-band (γ-PhC, γ-power), we defined the γ-bands for each animal and measure separately. For this purpose, we computed power and PhC-spectra in the period of MCs 2 and 3. The power spectra of each recording site were normalized by the mean power spectrum obtained during the baseline period of all trials in all conditions of that respective recording site, by first subtraction and then division by the baseline period's spectrum. Subsequently we averaged the normalized power spectra and PhC-spectra of all recording sessions and experimental conditions. The extent of the γ-band was determined based on the full width at half maximum of the mean PhC- and power-spectra respectively (Figure 2D, highlighted in gray).

The analysis of behavioral errors required pooling of trials across sessions. This excluded PhC as measure for synchronization since PhC requires a constant preferred phase difference across trials. This requirement might not be fulfilled for trials coming from different recording sessions. Therefore we used the magnitude squared coherence (MSC, see also: Carter et al., 1973) which can be computed within single trials and is subsequently averaged over trials. To obtain the MSC we first calculated the complex coherence values γ_*jk*_(*t, f*) between the signals of electrodes *j* and *k* at each time (*t*) and frequency (*f*) bin. This is achieved by multiplying for each trial the complex wavelet coefficient ${\tilde{x}}_{j}^{r}\left(t,f\right)$ with the complex conjugate of ${\tilde{x}}_{k}^{r}\left(t,f\right)$ to obtain the cross spectral density and normalizing it to the square root of the product of their auto spectral densities (again calculated by multiplying the complex wavelet coefficients with complex conjugates, but here of the same signal; complex conjugates are indicated by an overbar):

(5) $$\begin{array}{r} \gamma_{jk}\left(t,f,\;r\right)=\frac{{\tilde{x}}_{j}^{r}\left(t,f\right)*\bar{{\tilde{x}}_{k}^{r}\left(t,f\right)}}{\sqrt{\left({\tilde{x}}_{j}^{r}\left(t,f\right)*\bar{{\tilde{x}}_{j}^{r}\left(t,f\right)}\right)\left({\tilde{x}}_{k}^{r}\left(t,f\right)*\bar{{\tilde{x}}_{k}^{r}\left(t,f\right)}\right)}}. \end{array}$$

Subsequently, the absolute value of the complex coherence γ_*jk*_(*t, f*) is squared (Carter et al., 1973):

(6) $$\begin{array}{r} MSC\left(t,f,n\right)=\;{\left|\gamma_{jk}\left(t,f,\;n\right)\right|}^{2}. \end{array}$$

MSC-values were then averaged over trials. The time and frequency resolved MSC was bias-corrected by subtracting corresponding values derived from a shuffle predictor (Perkel et al., 1967; Gail et al., 2000). This shuffle predictor was obtained by computing 1000 times the MSC between ESA and LFP from randomly shuffled trials and averaging the results for each frequency bin. For computation of the MSC in the γ-band (γ-MSC) we defined a γ-band based on MSC spectra by the same procedure as described above for the γ-PhC.

### Experimental design and statistical analysis

This study includes physiological and behavioral data of two adult male macaque monkeys. The concept of the study demands two stimuli within the same pRF, each of them evoking a specific configuration of the local neuronal network in V4. As quickly accessible indicators for such a difference during the experimental sessions, we used response strength in firing rate and LFPs. We arranged position and color of the individual stimuli within the V4 pRF such that the response strengths were clearly different. To characterize the local processing of different behaviorally relevant stimuli with and without distractor within the V4 pRF, the task paradigm comprises five different conditions.

The two conditions with only one of the two possible stimuli presented inside the pRF are indicated by an “S” for single stimulus presentation, whereas the other conditions with both stimuli present are indicated by a “D” for double stimulus presentation. In the double conditions, each of the two stimuli could serve as the target of attention. In order to investigate whether processing of a relevant stimulus implies the same network configuration in absence as well as presence of a competing distractor, we required also in the single conditions attention to the stimulus within the pRF. This ensures a demand to configure a network for processing the same stimulus in corresponding single and double conditions.

Single and double conditions were labeled by a “+” or a “–” sign, depending on the stimulus in the pRF that was attended. Separately for each of the three measures (ESA-responses, ESA-LFP γ-PhC, and ESA-ESA γ-PhC) the labels “+” and “–” were assigned to the stimuli evoking the higher respectively lower values when presented alone. Thus, a stimulus inducing the stronger ESA-responses did not always also induce the higher γ-PhC (i.e., the same stimulus could be labeled “+” for ESA-responses and “–” for γ-PhC). For comparison of our ESA results to earlier work we also included a double condition with attention directed away from the pRF (D0). Together this results in five attentional conditions: S^+^, S^−^, D^+^, D^−^, and D^0^.

The rationale of the study required that the individual recording sites or site pairs had to fulfill the following criteria to be included in the analysis: (1) Recording sites had to be located in the upper cortical layers. (2) Neurons needed to respond significantly to each of the stimuli presented alone within the pRF, as measured by ESA-response for measures including ESA and by γ-LFP power for measures including LFP. (3) The values of ESA-responses or γ-PhC (depending on the analysis) for the two single stimulus conditions had to be sufficiently different to make sure the stimuli were driving the local population differently.

Criterion 1) was applied to avoid comparing of phase relations between neurons and overall population activity of different layers. Due to the typical recording procedure (lowering the electrode only until the first responses were found), this meant that recordings happened most likely in the upper layers. The location in the upper cortical layers was verified by the polarity of the evoked potential caused by stimulus onset (Schroeder et al., 1991). The significant activation (criterion 2) of neurons during the analysis window (MC 2/3, for explanation see below) was tested for ESA-values or LFP γ-power (depending on which measure was used for PhC estimation) against the respective values during the baseline period (starting after 150 ms (250 ms for monkey B) to its end; Wilcoxon signed-rank test, critical α-level: 0.05).

A sufficient difference of values during single conditions (criterion 3) was required for evaluating whether the ESA-response or γ-PhC observed in a double condition is more similar to the corresponding value observed in the S^+^ or in the S^−^ condition. Therefore, only sites or site pairs were considered, in which the analyzed measure differed by at least a factor of 1.33 between the two single conditions.

All analyses (with the exception of the error trial analysis) were performed within a time window comprising MCs 2 and 3 (Figure 2A, gray background). This analysis window was chosen because the target shape never appeared in MC 1, and at latest in MC 4. Therefore, attentional demands might be reduced during these periods. If the initial shape reappeared at the cued location at the end of MC 2 or 3, the time window ended 200 ms prior to the behavioral response. To exclude the potential survival of response related effects (as drescribed by Mirabella et al., 2007), we performed a control analysis with a cutoff period of 350 ms before the behavioral response. Neither for ESA-responses nor ESA-LFP γ-PhC we found differences of the sizes of effects or the level of significances. Individual values differed only marginally (on average by around 1%) as compared to our original values.

For quantitative analysis of the effect of attention on ESA-responses and γ-PhC across sessions and animals, the mean values for each of the five different attention conditions observed for a recording site or recording site pair were normalized by dividing them by the average of these five values. The statistical analysis was performed using non-parametric Wilcoxon signed-rank tests and Wilcoxon rank-sum tests at a critical α-level of 0.05. In case of multiple comparisons, all *p*-values were Bonferroni corrected, except for *p*-values that were already higher than the critical α-level of 0.05 before Bonferroni correction.

To investigate potential relations between neuronal activity patterns and behavioral performance, we compared ESA-responses and the synchronization of ESA with the LFP between correctly performed trials and trials terminated by a false alarm during MC 2/3. The selection criteria for contributing recording sites and site pairs were the same as described above. Because of their small number, the false alarm trials were pooled across sessions for each animal separately and MSC instead of PhC was used as a measure of synchronization (see above). For the analysis, a time window of 400 ms (monkey B: 500 ms) aligned to and ending 200 ms before the behavioral response was used. Thus, the duration of the time window was equivalent to half of a MCs' duration. For quantifying the differences between false alarms and correctly executed trials, 1000 randomly compiled sets of correctly performed trials were generated. For each set, we randomly selected the same number of correctly performed trials from each session as the session contributed false alarms. From these 1000 sets of correctly performed trials, the distributions of γ-MSC values were computed within the same time windows as for the corresponding false alarm trials in the corresponding sessions. The pooling procedure and random selection of trials was identical for investigating ESA-responses. Based on these distributions, we estimated the z-score values for γ-MSC and ESA-responses observed in the false alarm trials in comparison to correctly performed trials and derived corresponding confidence levels (probability derived from z–score chart).

## Results

We investigated attention-dependent changes of local network configuration in visual area V4 of two macaque monkeys, while the animals performed an attention-demanding shape tracking task (Taylor et al., 2005; Grothe et al., 2012). Briefly, animals had to covertly attend one of three or four stimuli with different shapes, colors and luminance, of which one was previously cued (Figure 1A). After static presentation, the shapes of all stimuli started morphing into other, randomly selected shapes, color, and luminance did not change (Figure 1B). Trials could contain up to four such MCs. Animals were required to detect the reappearance of the initial shape at the cued location. Animals broke fixation in 5.5% (monkey T) and 24.9% (monkey B) of trials respectively, the average performance disregarding fixation errors was 87.1 and 93.3% correct trials for monkey T (28 recording sessions) and B (34 recording sessions), respectively. In 6.6% (monkey T) and 3.8% (monkey B) of all trials (across all stimulus conditions), the response occurred before reappearance of the initial shape (false alarms), and in 6.3% (monkey T) and 2.9% (monkey B) the response occurred too late (misses). For trials with attention directed to a stimulus inside the pRF, the proportion of false alarms differed significantly for one animal between trials with one and two stimuli in the pRF (monkey T: with distractor in pRF 6.8%, without distractor 5.1%, z = 2.5504, *p* = 0.0108; monkey B: with distractor in pRF 2.0%, without distractor 2.2%, z = −0.561, *p* = 0.5748; Wilcoxon signed-rank test).

To investigate attention-dependent modulations of γ-PhC and ESA-responses within local V4 networks, we recorded simultaneously with two closely spaced microelectrodes (330 μm distance between shanks) from 118 recording sites in supragranular layers 2/3 (monkey T: 54, monkey B: 64). Data were gathered in 62 recording sessions (monkey T: 28, monkey B: 34). Based on manual mapping, we found 57 pairs with overlapping pRFs (monkey T: 26, monkey B: 31).

### Attention-dependent modulation of spiking activity

We first verified whether ESA-responses in our paradigm revealed a similar pattern of attentional modulation as described in previous studies based on single-unit firing rates (Moran and Desimone, 1985; Treue and Maunsell, 1996; Luck et al., 1997; Desimone, 1998; Reynolds et al., 1999; Treue and Martínez Trujillo, 1999; Lee and Maunsell, 2010; Ni et al., 2012). These studies showed consistently that when two stimuli are present in a RF, firing rates were modulated by attention to similar levels as for the attended stimulus presented alone. Without attention, firing rates were intermediate. Figure 2A shows an example for ESA-responses under different attentional conditions with either one (S^+^/S^−^) or two (D^+^/D^−^) stimuli inside the same pRF (cf. Methods for details of labeling task conditions). Due to our study design, ESA-responses were stronger during S^+^ conditions than during S^−^ conditions. When both stimuli were present in the pRF, the ESA-responses were in-between those for the single stimulus conditions and depended on the allocation of spatial attention. ESA-responses were stronger when the well activating stimulus was attended (D^+^) than when the less activating stimulus was attended (D^−^). Quantitative analysis of 80 recording sites (monkey T: 37, monkey B: 43) revealed significant differences between the two conditions with both stimuli present in the pRF (Figure 2B). ESA-responses were significantly larger during D^+^ (median 1.3) than during D^−^ conditions (median: 0.8; z = 7.293, Bonferroni-corrected *p* < 10^−11^, Wilcoxon signed-rank test). Thus, ESA-responses during double conditions were shifted toward the response levels induced when the attended stimulus was presented in isolation (S^+^: median: 1.3; S^−^: median: 0.6). Conditions which required the animals to direct attention away from the two stimuli inside the pRF to one of the stimuli located in the opposite hemi-field resulted in intermediate responses (D^0^: median 0.9). These responses were significantly different from the responses for the two other conditions with two stimuli in the pRF (D^+^/D^0^: z = 7.77, *p* < 10^−10^; D^−^/D^0^: *z* = −3.0504, *p* = 0.0069; *p*-values are Bonferroni corrected, Wilcoxon signed-rank test).

To quantify the extent of attentional modulation of responses in the double conditions and relate it to the difference between the responses caused by each of the two different stimuli alone we used an attentional modulation index (AMI). It is computed as the ratio of the attention-dependent difference between responses obtained in the D^+^ and D^−^ condition to the difference between responses in the S^+^ and S^−^ condition (AMI = ((D^+^ - D^−^) / (S^+^-S^−^))^*^100). The AMI reaches 100%, if attention modulated ESA-values such that the differences between double conditions on the one side and single conditions on the other side are equal. An AMI of zero indicates that there is no effect of attention when both stimuli are present. AMI values larger than 100% indicate that the difference of responses between D^+^ and D^−^ is even larger than between the single conditions. Negative values correspond to an opposite modulation in the double conditions as compared to the single conditions (D^+^ smaller than D^−^). For ESA-responses the median AMI value of 68.4% shows that the difference between D^+^ and D^−^ conditions is similar to that observed during the respective single conditions, but does not reach the same size. In summary, the attention-dependent modulation of ESA-responses under the stimulus and task conditions of the present experiments is well in line with previous findings on single-unit firing rates in areas V4 and MT.

### Attention-dependent changes of γ-PhC between neurons and overall population activity

After having confirmed the expected effect of selective attention on response strength, we tested our hypothesis that effective processing of an attended stimulus is associated with a specific configuration of functional connectivity within the local neuronal network. To this end, we compared the strength of γ-band synchronization between a small group of neurons (ESA) and the overall population activity (as measured by the LFP) either for a particular stimulus presented alone or together with a distractor in the pRF.

We calculated the PhC between ESA and LFP taken from two separate, closely spaced electrodes. In the following ESA-LFP PhC analysis, the designation of stimuli as “^+^” or “^−^” depended on the strength of γ-PhC induced by the two stimuli when presented alone. Figure 2C provides an example case of monkey B, showing the phase coupling between ESA and LFP in the γ-band (57.7 Hz to 86.8 Hz), persisting throughout MCs 2 and 3. The time averaged strength of the γ-PhC for the two conditions with only one stimulus in the pRF was 0.34 for the S^+^ and 0.13 for the S^−^ condition, indicating two configuration states that are separable with our network interaction proxy. When both stimuli were simultaneously present in the pRF and one of them was attended, the degree of synchronization closely matched the values of the corresponding singe stimulus condition (D^+^: mean 0.3; D^−^:.mean 0.15). With attention directed outside the pRF, the γ-PhC was intermediate (D^0^: mean 0.26).

Mean PhC-spectra for all recording site pairs confirmed the similarity of γ-PhC between conditions requiring to attend the same stimulus either in the presence or in absence of a distractor inside the pRF, for both animals (Figure 2D). A stimulus inducing low γ-PhC values when presented alone (S^−^) induced similarly low γ-PhC values even in the presence of a distractor inducing high γ-PhC when presented alone (D^−^). Conversely, when the stimulus inducing strong γ-PhC was attended, the level of γ-PhC stayed similarly high when a distractor inducing weak γ-PhC was present in the pRF (compare S^+^ and D^+^). The attention-dependent modulation of γ-PhC for all ESA-LFP pairs is shown in Figure 2E. The scattering of entries around the diagonal line indicates the similarity between the normalized γ-PhC values for attending a stimulus without versus with distractor in the pRF for both animals (*n* = 90). Note, that each recording site pair may deliver two ESA-LFP pairs since each electrode contributed an ESA and a LFP signal. The differences between normalized γ-PhC-values for the two conditions requiring to attend the stimulus inducing strong γ-PhC were small but significant (S^+^: median 1.42, D^+^: median 1.27, *z* = 4.6296, Bonferroni corrected *p* < 10^−4^, Wilcoxon signed-rank test). For conditions requiring to attend the stimulus inducing weak γ-PhC, the difference was not significant (S^−^: median 0.68, D^−^: median 0.63; *z* = 1.3017, *p* = 0.1930). In contrast, the difference between double conditions was large and highly significant (D^+^: median 1.27, D^−^: median: 0.63, *z* = 7.1481, Bonferroni corrected *p* < 10^−11^, Wilcoxon signed-rank test). The AMI, as a measure for the degree of attentional modulation in the double conditions as compared to the single conditions reached a median value of 81.8% and was significantly larger than the AMI for ESA-responses (ESA-responses: 68.4 %, *p* = 0.0142, *z* = −2.4523, Wilcoxon rank-sum test). This close match of γ-PhC between ESA and LFP signals for conditions requiring to attend the same stimulus (S^+^/D^+^ and S^−^/D^−^) also holds true for the individual animals (see Table 1). We found these significant modulations of PhC only in the γ-frequency range, but not for other frequency bands.

**Table 1:** Comparison of ESA-LFP γ-PhC values observed during different stimulus conditions for both animals.

**Compared conditions**	**Monkey T (*****n*** = **34)**		**Monkey B (*****n*** = **56)**	
S^+^/D^+^	1.48/1.54	*p* = 0.4675, z = 0.7266	1.41/1.18	*p* < 10^−7^*, z = 5.4408
D^+^/D^−^	1.54/0.55	*p* < 10^−3^*, z = 4.0433	1.18/0.63	*p* < 10^−8^*, z = 5.9791
D^−^/S^−^	0.55/0.51	*P* = 0.1909, z = 1.3079	0.63/0.78	*p* = 0.0027*, z = 3.3199

*The leftmost column describes the two task conditions that are compared. For each animal, the left column shows the normalized median γ-PhC values for these two conditions, whereas the right column provides the results of the corresponding Wilcoxon signed-rank tests. The asterisk indicates Bonferroni-corrected p-values. ESA-LFP γ-PhC values during ST-task for individual animals*.

To ensure that the stimulus specific differences of γ-PhC do not reflect very weak or lacking ESA and LFP oscillations, we examined the strength of underlying γ-oscillations. Supplementary Figures 1A and B show that not only during S^+^ and D^+^ conditions, but also during S^−^ and D^−^ conditions, γ-band oscillations were sufficiently large to ensure meaningful phase estimations for the PhC-measure. To investigate whether the modulations of γ-PhC simply reflect the modulations in ESA and/or LFP γ-power, we analyzed how well the reduction of γ-PhC between D^+^ and D^−^ conditions can be explained by changes in ESA and LFP γ-power (Supplementary Figures 1C,D). There were no significant correlations between the γ-PhC reduction and modulations of LFP or ESA γ-power (γ-PhC/ γ-LFP-power: Pearson's correlation, *r* = 0.1821, *p* = 0.11; γ-PhC/ γ-ESA-power: *r* = 0.17, *p* = 0.1252).

### Attention-dependent modulation of functional coupling between groups of neurons

**Figure 3:**
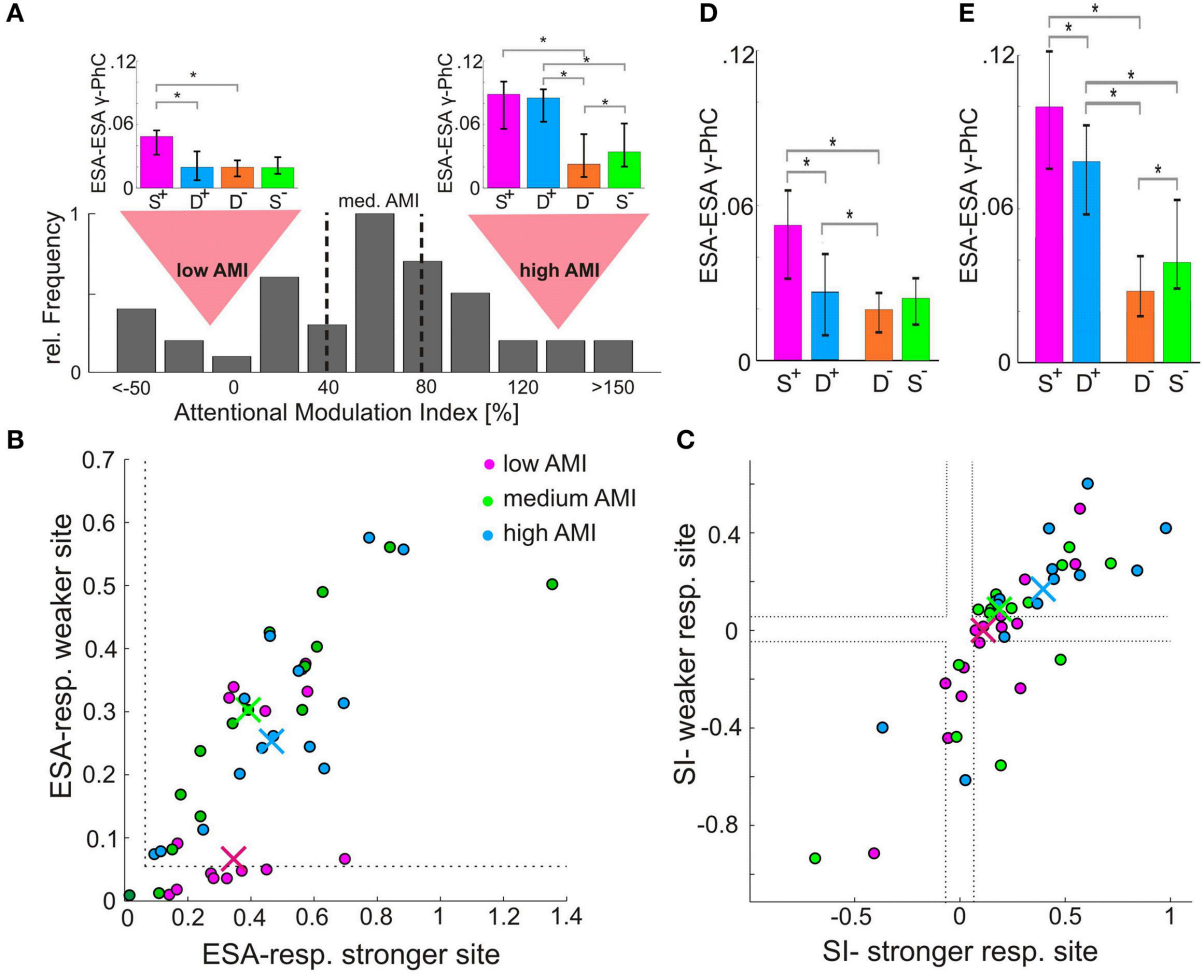
Dependence of ESA-ESA γ-PhC modulation on level of ESA-responses and stimulus specificity. **(A)** Distribution of AMI-values based on ESA-ESA γ-PhC (*n* = 44). The dashed vertical lines indicate borders between the third with lowest, the third with medium, and the third with highest AMI-values. The bar-plot insets above the distribution depict for the low and the high AMI group the median normalized γ-PhC between ESA-signals, which determine the AMI values. **(B)** Scatter-plot of normalized ESA-responses for the stimulus inducing higher γ-PhC between ESA-signals (S^+^ condition). An entry represents the ESA-responses of both recording sites contributing to the γ-PhC, with the site showing higher ESA-responses plotted on the X-axis. The different colors represent the high, medium, and low AMI-group, as illustrated in **(A)**. The median values for each group are depicted as correspondingly colored crosses. **(C)** Same as in **(B)**, but for the stimulus specificity of the sites constituting a pair. Sites showing higher SI-values are plotted on the X-axis. **(D)** Histogram of median normalized γ-PhC between ESA signals of the unspecific group (at least one site with ESA-responses below 0.05, see **B** or SI-values between −0.05 and 0.05 or with opposite stimulus preference, see **(C)** cases between dashed lines and in lower right quadrant). The error-bars indicate 95% confidence intervals. Asterisks indicate significant differences (*p* < 0.05) **(E)** same as in **D**, but for the specific group (pairs which were not classified as unspecific, see **D**).

Processing of different stimuli in a local network is thought to depend on different patterns of functional coupling strengths between its neurons. Therefore, we investigated whether the strength of functional connections between two small groups of neurons as measured by the ESA-ESA γ-PhC in the presence of two stimuli matched that observed for the attended stimulus presented alone. Here the designation of stimulus conditions as S^+^ or S^−^ was based on the strength of the ESA-ESA γ-PhC induced by the two different stimuli shown in these conditions. Contrary to our expectation, we found that the difference between double conditions (D^+^/D^−^) was much smaller than between single conditions (S^+^/S^−^) as reflected by a median AMI of 57.9% (*n* = 44). This raises the question whether this rather low AMI is characteristic for the γ-PhC between two subpopulations of neurons of the same population. The distribution of AMI values (Figure 3A) indicates that this is not the case since the AMI differed strongly over a wide range between pairs.

To investigate whether the wide range of AMI-values might be related to different response characteristics of the two constituting sites, we split the pairs according to their AMI value into three equally sized groups. (Figure 3A, dashed lines separate low, medium and high AMI group). First, we analyzed the relation between the pairs' AMI values for γ-PhC and the ESA-responses of their corresponding sites during S^+^ conditions. Almost half (47%) of the pairs of the low AMI-group (Figure 3B, red dots) had at least one site, which responded very weakly (ESA response strength < 0.05, border indicated by gray dashed lines in Figure 3B) to the stimulus inducing high γ-PhC. There were no such sites for the high AMI-group (blue dots) and only two pairs with such low responses for the medium group (green dots). Chi-square tests of independence confirmed, that there are significantly more such pairs in the low AMI group than in the high AMI-group (χ2 (1, *N* = 29) = 8.61, Bonferroni-corrected *p* < 0.01). All other comparisons between groups revealed no significant differences after Bonferroni correction (low AMI/medium AMI; medium AMI/high AMI; χ2 (1, *N* = 30) < 3.8, Bonferroni-corrected *p* > 0.05). Yet, the low-AMI group seems to consist of two groups of pairs, one with at least one site showing rather low responses (< 0.05) and another one where both sites showed normal response levels (values around 0.3–0.6). This may indicate that there are further combinations of response properties of a pair that go along with low AMI values.

Therefore, we analyzed whether the ratio between ESA-responses to the two stimuli might influence the AMI-values of γ-PhC as well. For each recording site a specificity index (SI) was calculated, which describes the degree of similarity (or dissimilarity) of the responses for the two different stimuli. The index reflects the difference between the ESA-responses during the S^+^ and the S^−^ conditions divided by their sum ((S^+^-S^−^)/(S^+^+S^−^)). Positive values indicate a preference for the stimulus of the S^+^ condition, negative values for the stimulus of the S^−^ condition and zero the same response strength for both stimuli. The scatterplot in Figure 3C depicts the SI-indices of the two contributing sites for each recording site pair of the three different AMI groups (high, medium and low values). A pair that has at least one site, which responds very similar to both stimuli, would be located between the dashed gray lines (Figure 3C; SI-values between −0.05 and 0.05). The entry of a pair with opposite stimulus preferences would be located in the lower right quadrant of the scatterplot (because the site with the higher SI-value is plotted on the X-axis). When comparing the distributions of entries it becomes obvious, that more entries of the low AMI group (red dots) are located between the gray dashed lines than for both other groups. Furthermore, there are no entries of the high AMI-group with opposite stimulus preference. Chi-square tests confirmed that the low AMI-group contained in comparison to the medium and the high AMI-group more pairs where at least one site shows almost no difference in responses to both stimuli (SI-values between −0.05 and 0.05) or even opposite stimulus preferences (high/low group: χ2(1, *N* = 29) = 10.208, Bonferroni-corrected *p* < 0.005; medium/low group: χ2(1, *N* = 30) = 6.53, Bonferroni-corrected *p* < 0.04). The corresponding differences between high and medium AMI group were not significant [χ2(1, *N* = 29) = 0.68, *p* = 0.41].

In summary, we found that high AMI-values correlate with a sufficient level of responses of both sites to the attended stimulus (Figure 3B). Furthermore, high AMI-values also correlate with higher levels of stimulus specificity for the same stimulus of the two constituting sites (Figure 3C). Thus, the weak synchronization in the D^+^ condition (Figure 3A, compare insets), which results in low AMI values, is observed in pairs with at least one site almost not responding to the attended stimulus (Figure 3B) or responding equally strong to both stimuli (Figure 3C). Such sites may therefore receive comparatively high proportions of signals related to the non-attended stimulus during the D^+^ condition. Strong synchronization between those neurons with a group of neurons processing mainly the attended stimulus could lead to a mixing of signals from target and distracter stimuli, which would counteract the enhanced and selective processing of the attended stimulus. It might therefore be beneficial that attention does not include those neurons into the ensemble processing the attended stimulus by enhancing their functional coupling.

In order to test whether such relations of response characteristics determine the different effects of attention γ-PhC, we split the 44 ESA-ESA pairs into two groups: One in which the promotion of distractor-related signals is unlikely and a second where this is more likely. Pairs were assigned to the first group (specific group) if both sites showed a sufficient response of at least 0.05 in the S^+^ condition and a preference for the same stimulus (both SI-values above +0.05 or both below −0.05). The remaining pairs were assigned to the second group (unspecific group).

The median γ-PhC values of the unspecific group (*n* = 24) are shown in Figure 3D, the corresponding γ-PhC values for the specific group (*n* = 20) in Figure 3E. The most evident difference between both groups is between the median γ-PhC values during D^+^-conditions. The unspecific group (Figure 3D) reached with a median value of 0.026 only 49.1% of the γ-PhC evoked during S^+^-conditions (median 0.053). For the specific group (Figure 3E), the γ-PhC during D^+^-conditions (median 0.08) reaches 80.4% of the value evoked by the S^+^-condition (median: 0.1). However, for both groups these differences were significant (unspecific group: *p* < 10^−3^, *z* = 3.9429; specific group: *p* = 0.0015, *z* = 3.6213, Wilcoxon signed-rank test, all *p*-values are Bonferroni corrected).

Another difference between specific (Figure 3E) and unspecific (Figure 3D) group can be observed when comparing D^+^ and D^−^ conditions. For the specific group, the difference was large and significant (D^+^: median 0.08, D^−^: median 0.027, Bonferroni corrected *p* < 10^−3^, *z* = 3.8826), whereas it was small, albeit significant for the unspecific group (D^+^: median 0.026, D^−^: median 0.021, Bonferroni corrected *p* = 0.036, *z* = 2.6857, both Wilcoxon signed-rank test). The γ-PhC of the unspecific group recorded during D^+^ conditions (median: 0.026) was not even significantly different from those recorded during S^−^ conditions (median 0.028; *p* = 0.3758, *z* = 0.8857, Wilcoxon signed-rank test). Yet, the same conditions evoked highly significant differences for pairs of the specific group (D^+^: median 0.08, S^−^: median 0.03; Bonferroni corrected *p* < 10^−3^, *z* = 3.8453, Wilcoxon signed-rank test). These differences in γ-PhC during double conditions explain the large and significant differences in AMI-values between both groups, with a median AMI of 84.7% for the specific group and only 29.9% for the unspecific group (*p* < 10^−5^, *z* = 4.0423, Wilcoxon signed-rank test). The AMI for the unspecific group was significantly lower than AMI-values for ESA-responses and ESA-LFP γ-PhC (ESA-responses/unspecific group: *p* < 10^−4^, *z* = −4.1392; unspecific group/ESA-LFP AMI: *p* < 10^−10^, *z* = −6.4883, *p*-values were Bonferroni-corrected). AMI-values for the specific group were significantly larger than those of ESA-responses and ESA-LFP γ-PhC (ESA-responses/specific group: *p* = 0.032, *z* = 2.4085; specific group/ESA-LFP AMI: *p* < 10^−8^, *z* = 5.9558, *p*-values were Bonferroni-corrected).

In summary, the attention-dependent modulation of γ-PhC between two neuronal sub-populations of the same local network depends on the response characteristics of their neurons for the two stimuli located in the pRF. The modulation is almost identical in conditions with and without distractor if both sub-populations respond sufficiently well to an attended stimulus and share the same stimulus preference. In contrast, if one of the two sub-populations shows only low responses to one of the stimuli, or the sites do not share th567e same stimulus preference, the strong attention-dependent difference between double conditions vanished.

### Network state and behavioral outcome

The results so far showed that attention modulates the pattern of γ-PhC in dependence of the attended stimulus with very similar values of γ-PhC in conditions with and without nearby distractor present. Hence, successful stimulus processing seems to depend on this specific pattern of synchronization within the local V4 network. If this holds true, the question arises, whether unsuccessful behavioral outcomes are associated with an incorrect pattern of γ-synchronization. Thus, we compared the γ-synchronization and spiking activity of periods directly preceding a behavioral error. A sufficient number of errors occurring during MC 2/3 for D^+^ and D^−^ conditions were available only for false alarms for both animals. Therefore we here show the false alarm trials pooled across all recording sessions for each animal individually. A comparison of misses and correctly executed trials for the monkey with a sufficient number of misses is shown in Supplementary Figure 2. The pooling across sessions made it necessary to investigate γ-band synchronization between ESA and LFP signals based on magnitude-squared coherence (cf. Materials and Methods for details). We analyzed γ-synchronization and ESA-responses within a time period of 400 ms for monkey T and 500 ms for monkey B. (Materials and Methods) terminating 200 ms before the behavioral response.

**Figure 4:**
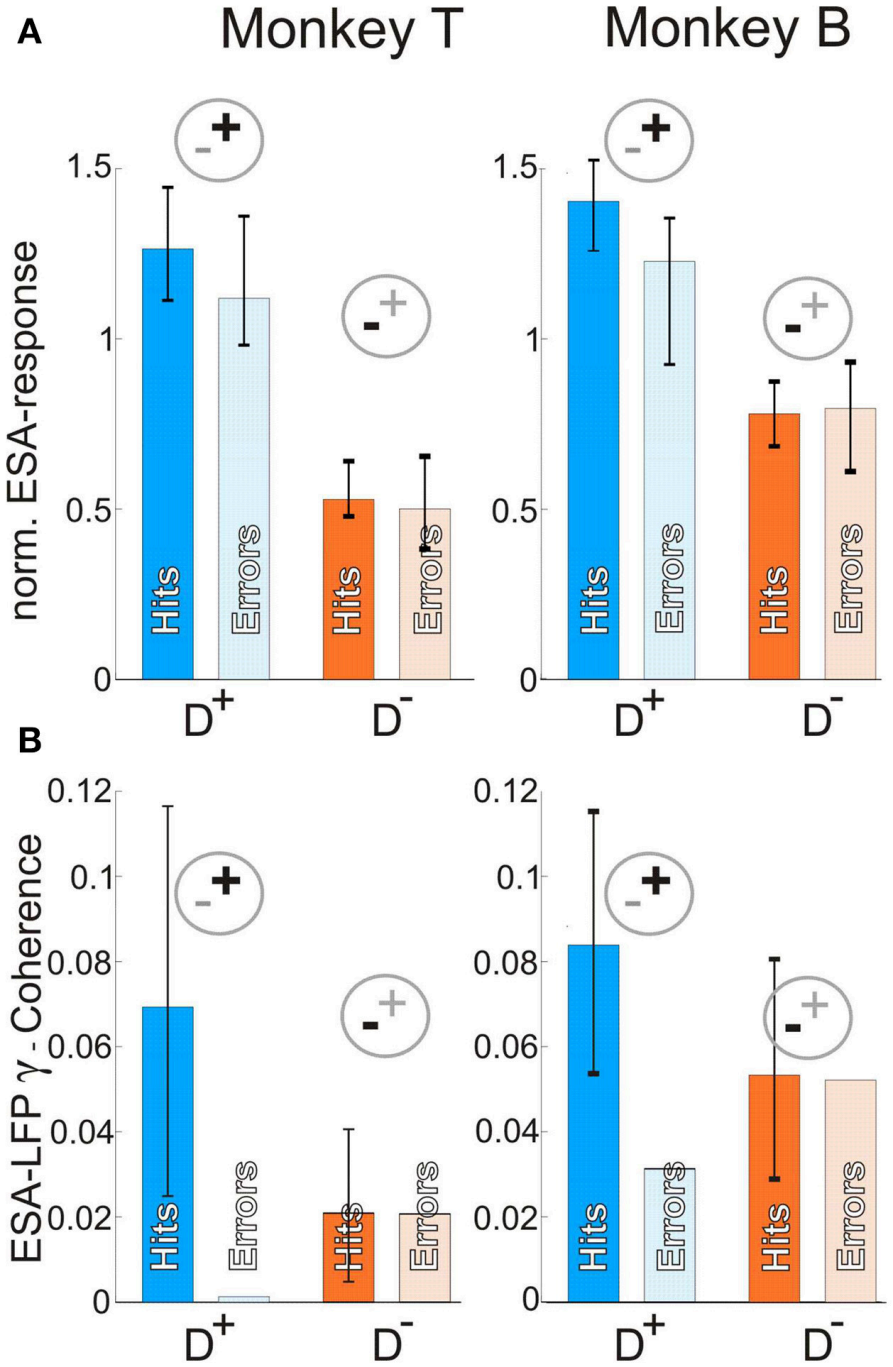
Neuronal correlates of different behavioral outcomes. **(A)** Median normalized ESA responses just before correct (dark blue and orange) and erroneous (light blue and orange bars) responses in trials requiring attention for one of the two stimuli within the pRF. Error-bars indicate 95% confidence intervals. **(B)** γ- coherence between ESA and LFP for the same conditions as in **(A)**. Note, that in contrast to *A e*rror-bars here indicate 95% of coherence values generated from 1,000 times compiling sets of correctly performed trials (c.f. Materials and Methods). The coherence value for error trials represents the coherence of all error trials pooled (due to small number). The value has to be interpreted with respect to the distribution of correctly performed trials and has no error-bars, since it is only one value. For abreviations and pictograms see Figures 1, 2.

The normalized ESA-responses were similar between periods preceding false alarms (Figure 4A, light blue and orange bars) and correct responses (Figure 4A, dark blue and orange bars) when attending the stimulus inducing stronger responses (D^+^) as well as for attending the stimulus inducing weaker responses (D^−^). There was no significant difference during D^+^ conditions for monkey T, and only small but significant differences for monkey B (monkey T: hits: 1.26, errors: 1.12, *n* = 52, z-score = −1.20; *p* = 0.12; monkey B: hits: 1.40, errors: 1.23, *n* = 39, z-score = −2.22, *p* = 0.013; z-transform, cf. section Materials and Methods). Similarly, the differences between ESA-responses in false alarm and correctly executed trials during D^−^ were not significant for both animals (monkey T: hits: 0.53, errors: 0.50, *n* = 108, z-score = −0.57; monkey B: hits: 0.78 errors: 0.80, *n* = 58, z-score = 0.26; *p* > 0.05 for both, z-transform).

In contrast to ESA-responses, the γ-synchronization between ESA and LFP (Figure 4B) showed a strong reduction for false alarm trials. During D^+^ conditions, the γ-synchronization was more than 98 % weaker for monkey T and still 62% weaker for monkey B in periods preceding a behavioral error as compared to correctly performed trials (monkey T: hits: 0.07, errors: 0.001, *n* = 28, *z*-value = −2.5083; monkey B: hits: 0.08, errors: 0.03, *n* = 38, *z*-value = −2.7822; *p* < 0.005 for both). During D^−^ conditions, the values between false alarm trials and correctly performed trials did not differ significantly (monkey T: hits: 0.02, errors: 0.02, *n* = 95, *z*-value = −0.0978, *p* = 0.46; monkey B: hits: 0.053, errors: 0.052, *n* = 38, *z*-value = −0.1123, *p* = 0.46). In summary, false alarms seem to correlate with a reduced level of γ-synchronization within the local neuronal network as compared to periods preceding correctly executed trials. In contrast, spiking activity does not reflect the erroneous behavioral responses, indicating the relevance of correctly configured networks for successful behavior.

## Discussion

Within the frame of this work, we examined whether the ability of neuronal networks to perform different functions on the same input could be implemented by an attention-dependent reconfiguration of the local neuronal networks. We hypothesized that this reconfiguration within the local neuronal networks is established by changing the pattern of functional connections between the network's neurons by adjusting the strength of their γ-synchronization. Thus, for effective processing of an attended stimulus, a stimulus-specific pattern of γ-synchronization would be necessary and patterns deviating from this specific configuration would result in a decreased stimulus processing.

Indeed, our results show that in the presence of two stimuli in the pRF, the γ-PhC between the spiking activity and the overall population activity within a local network depends on the attended stimulus and is highly similar to the values observed when the attended stimulus was presented alone in the pRF. The same holds true for γ-PhC of spiking activity between two groups of neurons of the same local network, but only when both responded sufficiently well to this stimulus and both shared the same stimulus preference. Correspondingly, the attentional modulation index (AMI) for ESA-LFP γ-PhC and ESA-ESA γ-PhC reached 82 and 85%. Similarly, ESA-responses were modulated during double conditions in the direction of levels observed for the attended stimulus presented alone, but to a lesser extent as indicated by the smaller AMI of 68%. Contrary to these qualitatively similar dependencies of γ-PhC and ESA-responses on the attended stimulus, both measures behaved qualitatively different prior to behavioral errors. ESA-responses in periods preceding false alarms were very similar to the responses observed prior to correctly terminated trials. In contrast, high levels of ESA-LFP γ-synchronization were strongly reduced in error trials. A similar pattern was observed for the comparison of misses and correctly executed trials shown for the monkey with a sufficient number of misses (SF 2). Furthermore, the γ-PhC between the ESAs of two groups of neurons where at least one site responded poorly to the attended stimulus, or very similar for both stimuli, strongly reduced when a distractor was added. These observations suggest that attention configures the pattern of functional coupling between the networks neurons specifically for effective processing of the relevant stimulus. Furthermore, our findings point to a top-down mechanism, which works in parallel to mechanisms gating relevant information to downstream areas and configures the synchronization within local network.

The results of attention-dependent modulations of spiking activity are well in line with previous work. Our task paradigm evoked different spiking activity for the two stimuli based on differences in location instead of orientation or motion direction as in previous studies. Furthermore, the stimuli were attended in conditions with and without distractor. Nevertheless the observed ESA-responses showed an attention-dependent modulation which was very similar to previous results (Moran and Desimone, 1985; Treue and Maunsell, 1996; Luck et al., 1997; Reynolds et al., 1999; Lee and Maunsell, 2010; Ni et al., 2012). Furthermore, a dependence of local synchronization on different stimulus configurations has been observed for anesthetized cats (Espinosa and Gerstein, 1988; Engel et al., 1991; Zhou et al., 2008) and for monkey under passive viewing conditions (Kreiter and Singer, 1996b; Frien et al., 2000; Maldonado, 2000). The latter results support the notion that processing of specific stimuli is associated with a specific pattern of synchronization within a local neuronal network, which is thought to reflect the functional coupling within this network.

However, we were interested in whether attention invokes stimulus-specific network configurations depending on the momentary behavioral demand, even though the stimulus input does not change. These attention-dependent changes would allow for an extensive number of different network configurations within the framework of the given anatomical connections by selectively modulating the strength of functional coupling between the network's neurons (Aertsen et al., 1989; Kreiter and Singer, 1996a; Fries, 2005; Kreiter, 2006; Gregoriou et al., 2009; Battaglia et al., 2012; Palmigiano et al., 2017). If such a specific pattern of γ-synchronization within a local network is crucial for its ability to process a specific, behaviorally relevant stimulus, the pattern should change as attention switches between stimuli. Furthermore, it should be very similar to the pattern observed in the absence of the distractor stimulus. Our results confirm this prediction. When instead of a single stimulus two closely spaced stimuli provided input signals to a local neuronal network in V4, the γ-PhC depended on which stimulus was attended and was very similar to the values observed when this stimulus was presented alone. The AMI of 85% for the γ-PhC between ESA of two groups of neurons of the same local network illustrates the high precision by which selective attention adjusts the functional coupling strengths when the same stimulus is attended. The significantly weaker AMI for the ESA-responses support the notion that response strength and γ-PhC are not trivial consequences of each other, but reflect different aspects of neural processing (Bichot et al., 2005; Buffalo et al., 2011).

The hypothesis of attention-dependent dynamic network configuration by γ-band synchronization is further supported by pairs, which do not preserve their high γ-PhC for an attended stimulus when a distractor stimulus is added. Of those pairs, at least one site responds either very weakly to the attended stimulus or relatively strong to the distractor stimulus. These neurons are likely to receive a comparably high proportion of input signals representing the distractor, since even an attention-dependent input gating mechanism suppresses distractor related signals only to a limited extent. The reduced strength of functional coupling between those neurons and neurons of the dynamically defined network processing the attended stimulus should therefore help to avoid interference of distractor signals with processing of the attended stimulus. If in contrast only one stimulus is present, even weakly driven neurons can contribute to a network processing this stimulus since they carry no signals of distractors that could interfere. Well in line Vinck et al. (2013) gave evidence for a decoupling of those neurons from the processing network, which provided only poor information about an attended stimulus, even though no nearby distractor was present.

The relevance of γ-synchronization for the functional configuration of the local neuronal network in V4 is further supported by the characteristics of neuronal activity directly preceding behavioral errors. While the attention-dependent modulation of ESA-responses were almost unchanged in comparison to correctly executed trials, strong γ-synchronization between groups of neurons and the local population activity (LFP) in correctly executed trials vanished before an error. Several other studies also reported correlations between behavioral performance and oscillatory power or synchronization as well as firing-rates. In contrast to our study, they either used a task with only one stimulus in the RF to investigate error-dependent differences of synchronization (Tallon-Baudry et al., 2004), or compared conditions with attention directed into the RF versus away from the RF (Womelsdorf et al., 2006; Gregoriou et al., 2014). The latter two studies report that firing rates and synchronization show qualitatively similar differences when comparing either slow and fast reaction times (Womelsdorf et al., 2006) or error-dependent changes (Gregoriou et al., 2014). The level of synchronization and the firing rates were higher during fast trials as compared to slow trials and during correctly executed trials when compared to erroneously terminated trials. However, for our specific behavioral paradigm, recording constellation, and the attentional conditions compared here (attention directed always to a stimulus within the pRF of the recorded V4 population), we observed qualitatively different results for firing rates and γ-synchronization.

To put these results into perspective, we briefly recapitulate the selective gating of relevant information between and within visual areas. Previous work showed that V4 neurons synchronize selectively with afferent V1 neurons representing the attended stimulus while desynchronizing with those representing distractors (Bosman et al., 2012; Grothe et al., 2012). Furthermore, we showed that mainly signals carrying specific signatures related to the attended stimulus enter into the local processing network in V4.(Grothe et al., 2018). These findings, together with theoretical investigations, point toward an attention-dependent routing mechanism based on highly selective changes of functional coupling between V4 neurons and different subsets of their afferent inputs (Börgers and Kopell, 2008; Masuda, 2009; Tiesinga and Sejnowski, 2010; Battaglia et al., 2012; Hahn et al., 2014; Harnack et al., 2015). The almost unchanged level of firing rates during error and correctly performed trials in both task conditions, as shown in Figure 4A, indicate that attention was correctly directed to the target stimulus: If monkeys had attended the nearby distractor in the same pRF, the firing rates should have approached the strength associated with the distractor. A similar consideration holds if the animals would have allocated attention elsewhere. In this case, one would expect an intermediate firing rate, as observed for trials with attention directed away from the pRF (D^0^-condition, Figure 2B). Our findings therefore indicate that those attention-dependent top-down mechanisms that selectively route the signals of the attended stimulus from upstream areas to the V4 neurons, were unlikely the source of the error.

The degradation of local γ-synchronization preceding errors rather indicates that successful processing of an attended stimulus also depends on a specific synchronization in the supragranular layers of V4. The recorded neurons in the upper layers of V4 show the expected level of spiking activity and are therefore likely to receive the correctly selected signals from the granular layer of V4. Thus, their local γ-synchronization is unlikely to depend solely on successful gating of the afferent stimulus related signals to V4. Rather, it is subject to attention-dependent top-down mechanisms, independent of a gating mechanism for the afferent bottom-up input to V4. In line with our findings, Vinck and Bosman (2016) concluded in a recent review based on experimental data, that γ-oscillations in superficial and in granular layers can be generated fairly independent of each other. Thus, our observed strong degradation of γ-synchronization within the local network in superficial layers during error trials, might arise from a failure of attention-dependent top-down mechanisms to synchronize the neuronal network in layer 2/3. At the same time, attentional mechanisms are still successful in selective gating of relevant information to layer 4 neurons. However, since neurons located in supragranular layers provide the cortico-cortical output projections, the disappearance of their strong γ-synchronization results in a reduced impact of their spikes on down-stream neurons (Niebur et al., 1993). Thus, the signal-to-noise ratio of the behaviorally relevant signal, and therefore its processing further downstream, is expected to be compromised. This may well give rise to erroneous responses (Taylor et al., 2005; Womelsdorf et al., 2006; Martin and von der Heydt, 2015) and might explain the different correlations between local γ-synchronization and firing-rates with behavioral outcome.

## Conclusion

In summary, our results show clear similarities as well as specific differences between the attention-dependent modulation of γ-synchronization and spiking activity in local neuronal networks of area V4. These results are well in line with the expectations for an attention-dependent mechanism that structures functional coupling strengths and hence the functional configuration of a local network by modulating γ-band synchronization. We conclude that: (1) Attention adjusts the pattern of functional coupling strengths within a local neuronal network specifically for processing of an attended stimulus. (2) Attention dynamically decouples neurons from a network processing the attended stimulus when they would compromise processing with additional distractor related signals. (3) Degraded synchronization within the local network occurs just before behavioral errors in spite of almost intact attention-dependent firing-rates, indicating an error-location beyond afferent signal gating.

## Author contributions

AK, SM, and IG designed the experiment. ED and MH performed the experiment and analysis. ED and AK wrote the manuscript. SM, MH, and IG discussed and reviewed the manuscript.

### Conflict of interest statement

The authors declare that the research was conducted in the absence of any commercial or financial relationships that could be construed as a potential conflict of interest.
