# Supplementary material for: Attention Configures Synchronization Within Local Neuronal Networks for Processing of the Behaviorally Relevant Stimulus
Source: Front Neural Circuits. 2018 Aug 29;12:71. doi: 10.3389/fncir.2018.00071 (PMC6123385; doi:10.3389/fncir.2018.00071)
Supplement: Supplementary file 1 [file Data_Sheet_1.DOCX]

Supplementary Material

Attention-Dependent Dynamic Gamma-Band Synchronization as a Mechanism for Local Network Configuration in Monkey Area V4

**Eric Drebitz^*^, Marcus Haag, Iris Grothe, Sunita Mandon, Andreas Kurt Kreiter**

*** Correspondence:** Eric Drebitz: drebitz@brain.uni-bremen.de

#
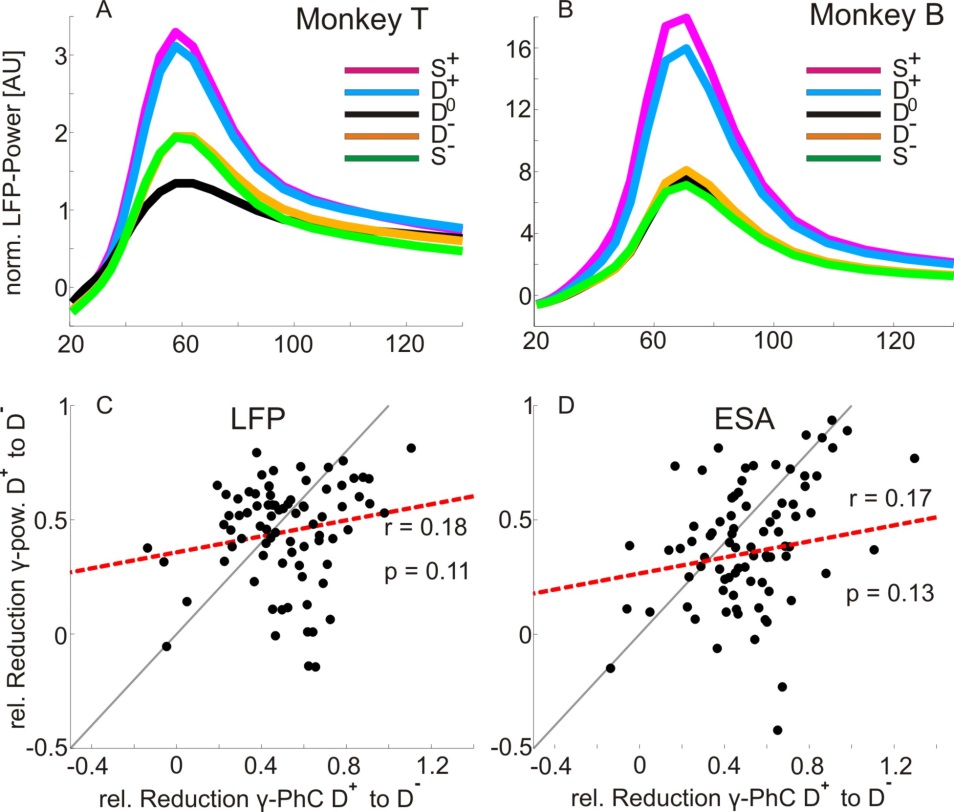
Supplementary Figures

**Supplementary Figure 1. A,** Average spectrum of normalized LFP-power during MCs 2/3 for the 5 task conditions of all recording sessions for monkey T. ***B,*** same as in *A* but for monkey B. **C*,*** Scatterplot of attention-dependent relative reduction of ESA-LFP γ-PhC from D+ to D- vs. the relative reduction of LFP γ-power between these conditions. The gray line is the line of identity, the red dashed line the regression line. **D*,*** same as in E, but for comparison ESA-LFP γ-PhC vs. ESA γ-power.


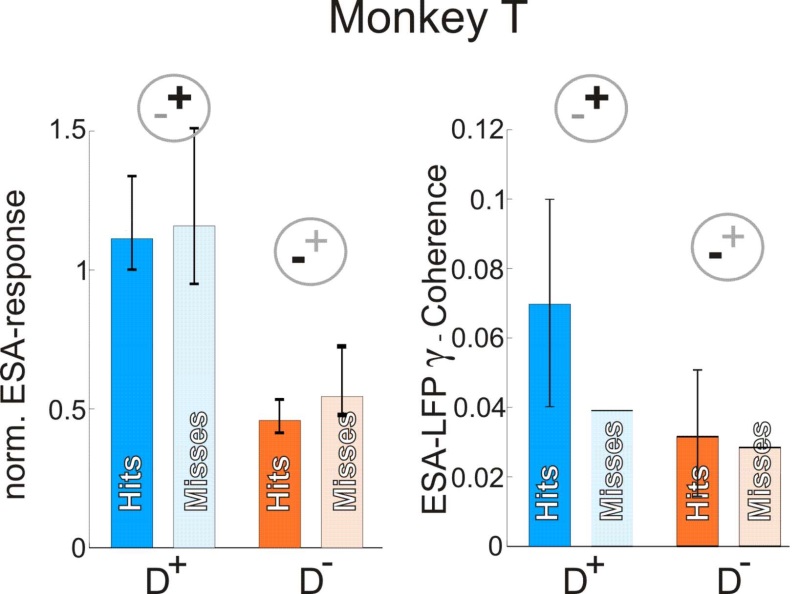


**Supplementary Figure 2.** Neuronal correlates of different behavioral outcomes. ***Left***, Median normalized ESA responses just before correct (dark blue and orange) and erroneous (light blue and orange bars, misses) responses in trials requiring attention for one of the two stimuli within the pRF. Error-bars indicate 95% confidence intervals. ***Right,*** same as for left subplot but for γ- synchornization between ESA and LFP, Note, that *e*rror-bars here indicate 95 % of coherence values generated from 1000 times compiling sets of correctly performed trials (c.f. Materials and Methods). The synchronization value for error trials represents the coherence of all error trials pooled

**Supplementary Results**

Neuronal correlates and behavioral outcome for misses vs. hits

Due to the small number of trials where monkey B failed to detect the reappearance of the target shape (misses), the comparison between misses and correctly executed trials was restricted to monkey T. In contrast to false alarms, there is no behavioral response to which the analysis could be triggered. We therefore compared the time period representing the second half (400 ms) of the morph cycle in which the target shape became apparent, to the same period of correctly terminated trials (ending 200 ms prior to the behavioral response). The normalized ESA-responses for conditions which required attending the stimulus inducing stronger responses (D^+^) were not significantly different between hits and misses (hits: 1.16, misses: 1.15, n= 68, z-score = 0.3745; p = 0.355, z-transform, cf. Materials and Methods). There was a small and significant difference between misses and correctly executed trials for double conditions requiring to attend the stimulus inducing weaker responses (D^-^) with higher ESA-responses for misses (hits: 0.46, misses: 0.54, n= 108, z-score = 1.82; p = 0.034, z-transform). In contrast, the magnitude of γ-synchronization decreased by 43 % for misses as compared to correctly executed trials during D^+^ conditions. This difference was significant (hits: 0.07, misses: 0.04, n= 49, z-score = -1.65; p = 0.0495, z-transform), whereas there was no significant difference between misses and hits for D^-^ conditions (hits: 0.03, misses: 0.0288, n = 103, z-score: -0.3659, p = 0.3557).
